# Serum magnesium levels and risk of coronary artery disease: Mendelian randomisation study

**DOI:** 10.1186/s12916-018-1065-z

**Published:** 2018-05-17

**Authors:** Susanna C. Larsson, Stephen Burgess, Karl Michaëlsson

**Affiliations:** 1 Unit of Nutritional Epidemiology, Institute of Environmental Medicine, Karolinska Institutet, 171 77 Stockholm, Sweden; 2 MRC Biostatistics Unit, University of Cambridge, Cambridge, UK; 3 Department of Public Health and Primary Care, University of Cambridge, Cambridge, UK; 4 Department of Surgical Sciences, Uppsala University, Uppsala, Sweden

**Keywords:** Coronary artery disease, Magnesium, Mendelian randomisation, Single-nucleotide polymorphisms

## Abstract

**Background:**

Observational studies have shown that serum magnesium levels are inversely associated with risk of cardiovascular disease, but whether this association is causal is unknown. We conducted a Mendelian randomisation study to investigate whether serum magnesium levels may be causally associated with coronary artery disease (CAD).

**Methods:**

This Mendelian randomisation analysis is based on summary-level data from the CARDIoGRAMplusC4D consortium’s 1000 Genomes-based genome-wide association meta-analysis of 48 studies with a total of 60,801 CAD cases and 123,504 non-cases. Six single-nucleotide polymorphisms associated with serum magnesium levels at genome-wide significance were used as instrumental variables.

**Results:**

A genetic predisposition to higher serum magnesium levels was inversely associated with CAD. In conventional Mendelian randomisation analysis, the odds ratio of CAD was 0.88 (95% confidence interval [CI] 0.78 to 0.99; *P* = 0.03) per 0.1-mmol/L (about 1 standard deviation) increase in genetically predicted serum magnesium levels. Results were consistent in sensitivity analyses using the weighted median and heterogeneity-penalised model averaging methods, with odds ratios of 0.84 (95% CI 0.72 to 0.98; *P* = 0.03) and 0.83 (95% CI 0.71 to 0.96; *P* = 0.02), respectively.

**Conclusions:**

This study based on genetics provides evidence that serum magnesium levels are inversely associated with risk of CAD. Randomised controlled trials elucidating whether magnesium supplementation lowers the risk of CAD, preferably in a setting at higher risk of hypomagnesaemia, are warranted.

**Electronic supplementary material:**

The online version of this article (10.1186/s12916-018-1065-z) contains supplementary material, which is available to authorized users.

## Background

Magnesium is the second most abundant intracellular cation. It plays a crucial role in many processes regulating cardiovascular function, such as vascular tone, endothelial function and myocardial excitability, and it is involved in regulation of glucose and insulin metabolism [1, 2]. Experimental evidence indicates that magnesium insufficiency promotes atherosclerosis and that magnesium fortification attenuates atherogenesis [2–7]. Moreover, randomised controlled trials have shown that magnesium supplementation improves endothelial function [8, 9] and reduces blood pressure [8, 10–12], arterial stiffness [13], fasting glucose [12, 14], insulin resistance [15] and postoperative arrhythmias [16, 17]. Randomised controlled trials assessing whether magnesium supplementation may prevent cardiovascular events are lacking.

Evidence from observational studies indicates that high circulating magnesium levels and magnesium intake are associated with a modest reduction in risk of cardiovascular disease, including coronary heart disease [18, 19], but the causality of these associations is unknown. The observed inverse association between magnesium and cardiovascular disease may be due to confounding by other potentially cardioprotective nutrients in magnesium-rich foods or by health behaviours adopted by individuals consuming these foods. Rich food sources of magnesium include green leafy vegetables, legumes, nuts, seeds, avocados, dark chocolate, whole grains, yoghurt and fish. It has been estimated that magnesium intake from a normal Western diet is often inadequate. In the USA, two-thirds of the adult population has a magnesium intake below the estimated average requirement [20].

**Fig. 1:**
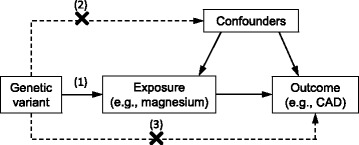
Diagram of the instrumental variables assumptions for Mendelian randomisation. The three assumptions are: (1) the genetic variant must be robustly associated with the exposure; (2) the genetic variant should not be associated with confounders of the exposure-outcome association; and (3) the genetic variant must influence the outcome through the exposure only and not through any direct or alternative pathways. The *dashed lines* represent pathways that violate the assumptions. *CAD* coronary artery disease

Exploiting genetic variants as instrumental variables of an exposure can strengthen causal inference regarding an exposure-outcome relationship. This technique, known as Mendelian randomisation (MR), reduces confounding because genetic variants are randomly allocated at meiosis and thus should be unrelated to self-selected lifestyle factors and behaviours. It also overcomes reverse causation bias since allelic randomisation always precedes the onset of disease. Causal inference from an MR study relies on the instrumental variable assumptions, which require that the genetic variant is robustly associated with the exposure; independent of confounders of the exposure-outcome relationship; and influences the outcome through the exposure only and not through any alternative causal pathway (Fig. 1) [21].

We applied a two-sample MR framework to determine the causal association between serum magnesium levels and coronary artery disease (CAD).

## Methods

### Genetic variants and data sources

We used an MR study design based on publicly available summary-level data from genome-wide association studies (GWASs) (Table 1). As instrumental variables for the MR analyses, we selected all single-nucleotide polymorphisms (SNPs) associated with serum magnesium levels at genome-wide significance (*P* < 5×10^− 8^) in the largest available GWAS on serum magnesium levels [22]. We selected all six SNPs that achieved genome-wide significance in the joint analysis of the discovery (*n* = 15,366 individuals) and replication (*n* = 8463 individuals) cohorts [22]. All the SNPs were in different genomic regions and in linkage equilibrium.

**Table 1:** Details of studies and datasets used for analyses

Exposure/outcome	Consortium	Participants	Web source if publicly available
Serum magnesium	CHARGE and replication studies [22]	23,829 individuals of European ancestry	Not available
Coronary artery disease	CARDIoGRAMplusC4D consortium’s 1000 Genomes-based GWAS [23]	184,305 individuals (60,801 CAD cases and 123,504 non-cases) of mainly European (77%) and Asian (19%) ancestry	www.cardiogramplusc4d.org/
Blood pressure	ICBP [29]	69,395 individuals of European ancestry	www.ncbi.nlm.nih.gov/projects/gap/cgi-bin/study.cgi?study_id=phs000585.v1.p1
Lipids	GLGC [30]	188,577 individuals of European ancestry	csg.sph.umich.edu/abecasis/public/lipids2013/
Glycaemic traits	MAGIC [31]	46,186 non-diabetic individuals of European ancestry	www.magicinvestigators.org/
Body mass index	GIANT [32]	339,224 individuals of mainly European (95%) ancestry	portals.broadinstitute.org/collaboration/giant/index.php/GIANT_consortium
Waist-to-hip ratio	GIANT [33]	224,459 individuals of mainly European (94%) ancestry	portals.broadinstitute.org/collaboration/giant/index.php/GIANT_consortium
Smoking	TAGC [34]	74,053 individuals of European ancestry	www.med.unc.edu/pgc/results-and-downloads

*CHARGE* Cohorts for Heart and Aging Research in Genomic Epidemiology Consortium, *GIANT* Genetic Investigation of Anthropometric Traits, *GLGC* Global Lipids Genetics Consortium, *ICBP* International Consortium for Blood Pressure, *MAGIC* Meta-Analyses of Glucose and Insulin-related traits Consortium, *TAGC* Tobacco and Genetics Consortium

Summary-level data (beta coefficients and standard errors) for the associations of the six magnesium-associated SNPs with CAD were acquired from the CARDIoGRAMplusC4D consortium’s 1000 Genomes-based genome-wide association meta-analysis of 60,801 CAD cases and 123,504 non-cases from 48 studies [23]. One SNP (rs7965584) was not part of the CARDIoGRAMplusC4D dataset and was replaced by a linked SNP (rs10858938; *r*^2^ = 0.96 in Europeans). In the CARDIoGRAMplusC4D consortium, CAD was defined using a broad definition that included myocardial infarction (about 70% of the total number of cases), acute coronary syndrome, chronic stable angina or coronary artery stenosis of at least 50% [23]. Ethical approval was not sought, because this study involved analysis of publicly available summary-level data (beta coefficients and standard errors) from GWASs, and no individual-level data were used.

### Statistical analysis

The main analysis was conducted using the conventional inverse-variance weighted method [24] (hereafter referred to as conventional MR analysis). Several sensitivity analyses were carried out, including (1) the leave-one-out analysis, in which one SNP in turn was removed to evaluate the impact of outlying SNPs; (2) the weighted median method, which gives accurate estimates if at least 50% of the instrumental variables are valid [24]; (3) the heterogeneity-penalised model averaging method, which provides consistent estimates if a plurality of the instrumental variables are valid [25]; and (4) MR-Egger regression, which can detect and adjust for pleiotropy [24, 26]. MR-Egger is disposed to effect estimate dilution due to the NO Measurement Error (NOME) assumption for the instrument-exposure associations. The NOME assumption was tested using the *I*^2^_GX_ statistic, and the MR-Egger estimate was adjusted for dilution using the simulation extrapolation (SIMEX) method [27]. The strength of the instrumental variables was assessed using the *F*-statistic [28].

To investigate potential pleiotropy and mediating pathways from serum magnesium to CAD, we performed conventional MR analyses of the association of serum magnesium levels with cardiometabolic risk factors, using publicly available GWAS data [29–34] (Table 1).

All reported odds ratios (ORs) with their 95% confidence intervals (CIs) are scaled to a 0.1-mmol/L (about one standard deviation [SD]) increase in serum magnesium levels. All statistical tests were two-sided and considered statistically significant at *P* < 0.05. The analyses were conducted using the mrrobust [35] and MendelianRandomization [36] packages.

## Results

**Fig. 2:**
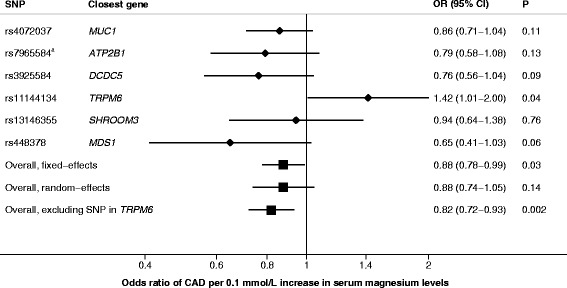
Association between genetically predicted serum magnesium levels and coronary artery disease. Odds ratios are scaled to a genetically predicted 0.1-mmol/L (about one SD) increase in serum magnesium levels. Analysis was conducted using inverse-variance weighted meta-analysis with standard errors calculated using fixed-effects or random-effects weights. *P*_heterogeneity_ between estimates from individual SNPs was 0.06 in analysis including all six SNPs and 0.74 in analysis excluding the outlying SNP in the *TRPM6* gene. ^a^Proxy (rs10858938) was used in the coronary artery disease data. *CAD* coronary artery disease, *CI* confidence interval, *OR* odds ratio

The six magnesium-associated SNPs explained 1.62% of the variance in serum magnesium levels, and the mean *F*-statistic was 64 (Table 2). Five of the SNPs were inversely, albeit non-statistically significantly, associated with CAD (Table 2). In conventional MR analysis, genetically predicted serum magnesium was inversely associated with CAD, but there was evidence of heterogeneity between estimates from individual SNPs (*P*_heterogeneity_ = 0.06). The ORs of CAD per a 0.1-mmol/L (about one SD) increase in genetically predicted serum magnesium levels were 0.88 (95% CI, 0.78–0.99; *P* = 0.03) and 0.88 (95% CI, 0.74–1.05; *P* = 0.14) when standard errors were calculated using fixed-effects and random-effects models, respectively (Fig. 2). In the leave-one-out analysis, it was found that rs11144134 in the *TRPM6* gene region was responsible for the heterogeneity among estimates from individual SNPs. After exclusion of this SNP, there was no heterogeneity between estimates (*P*_heterogeneity_ = 0.73), and the OR was 0.82 (95% CI, 0.72–0.93; *P* = 0.002) in both fixed-effects and random-effects models (Fig. 2).

**Table 2:** Characteristics of the single-nucleotide polymorphisms associated with serum magnesium levels

							Association with magnesium^a^			Association with CAD^a^		
SNP	Closest gene	Chr	EA^b^	EAF^c^	% variance explained	*F*-statistic	Beta (mmol/L)	SE	*P*	Beta^d^	SE	*P*
rs4072037	*MUC1*	1	T	0.54	0.57	136	0.010	0.001	2.0 × 10^−36^	−0.015	0.010	0.11
rs7965584^e^	*ATP2B1*	12	A	0.71	0.25	60	0.007	0.001	1.1 × 10^−16^	−0.016	0.011	0.13
rs3925584	*DCDC5*	11	T	0.55	0.25	60	0.006	0.001	5.2 × 10^−16^	−0.016	0.010	0.09
rs11144134	*TRPM6*	9	C	0.08	0.23	55	0.011	0.001	8.2 × 10^−15^	0.039	0.019	0.04
rs13146355	*SHROOM3*	4	A	0.44	0.19	45	0.005	0.001	6.3 × 10^−13^	−0.003	0.010	0.76
rs448378	*MDS1*	3	A	0.53	0.13	30	0.004	0.001	1.3 × 10^−8^	−0.017	0.009	0.06

*CAD* coronary artery disease, *Chr* chromosome, *EA* effect allele, *EAF* effect allele frequency, *SE* standard error, *SNP* single-nucleotide polymorphism

^a^Beta coefficients and standard errors were obtained from genome-wide association studies on serum magnesium (23,829 individuals) [22] and CAD (60,801 cases and 123,504 non-cases) [23]

^b^Allele associated with higher serum magnesium levels

^c^Frequency of the magnesium-raising allele in the magnesium genome-wide association study [22]

^d^Log odds ratio of CAD for each additional magnesium-increasing allele

^e^Proxy (rs10858938; *r*^2^ = 0.96 in European descent individuals) was used in the CAD data

Results were consistent in sensitivity analyses using the weighted median (OR, 0.84; 95% CI, 0.72–0.98; *P* = 0.03) and heterogeneity-penalised model averaging (OR, 0.83; 95% CI, 0.71–0.96; *P* = 0.02) methods (Additional file 1: Table S1). The MR-Egger analysis did not provide evidence of either directional pleiotropy (intercept –0.023; *P* = 0.21) or a causal association (OR = 1.19; 95% CI, 0.72–1.98; *P* = 0.50), but the precision of the estimates was low (Additional file 1: Table S1). *I*^2^_GX_ was 0.87 (relative bias of 13% towards the null), and adjusting for dilution bias using the SIMEX method did not materially change the MR-Egger estimate (Additional file 1: Table S1).

In conventional MR analyses, genetic predisposition to higher serum magnesium levels was weakly associated with higher systolic blood pressure (*P* = 0.04) and triglycerides (*P* = 0.04), but was not associated with diastolic blood pressure, cholesterol, fasting glucose, fasting insulin, insulin resistance, body mass index, waist-to-hip ratio or smoking (Table 3).

**Table 3:** Associations between genetically predicted serum magnesium levels and cardiometabolic risk factors

Outcome	Estimate^a^	*P* value
Continuous outcomes	Beta (95% CI)	
Diastolic blood pressure	0.46 (−0.34 to 1.26) mm Hg	0.26
Systolic blood pressure	1.31 (0.05 to 2.57) mm Hg	0.04
Low-density lipoprotein cholesterol	0.06 (−0.00 to 0.13) SD	0.07
High-density lipoprotein cholesterol	−0.03 (−0.09 to 0.03) SD	0.34
Triglycerides	0.06 (0.00 to 0.12) SD	0.04
Fasting glucose	0.02 (−0.03 to 0.07) mmol/L	0.35
Fasting insulin	0.02 (−0.03 to 0.07) log pmol/L	0.43
HOMA-IR	0.01 (−0.04 to 0.06)	0.67
BMI	−0.02 (−0.07 to 0.02) SD	0.34
Waist-to-hip ratio adjusted for BMI	0.02 (−0.03 to 0.07) SD	0.50
Cigarettes per day	−0.59 (−1.66 to 0.49) cigarettes/day	0.29
Binary outcomes	OR (95% CI)	
Ever smoker	1.00 (0.98 to 1.01)	0.62
Former smoker	1.00 (0.98 to 1.02)	0.78

*BMI* body mass index, *CI* confidence interval, *HOMA-IR* homeostatic model assessment of insulin resistance, *OR* odds ratio, *SD* standard deviation

^a^Estimates correspond to a 0.1-mmol/L (about one SD) increase in genetically predicted serum magnesium levels

## Discussion

**Fig. 3:**
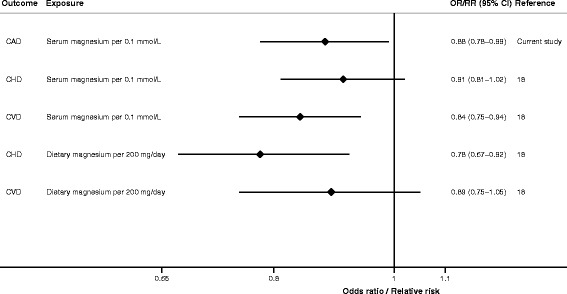
Associations of serum magnesium and magnesium intake with CAD, coronary heart disease and cardiovascular disease. The summary results are from the current Mendelian randomisation study of genetically predicted serum magnesium levels in relation to CAD and a previous meta-analysis of observational prospective studies of serum magnesium levels and dietary magnesium intake in relation to coronary heart disease and cardiovascular disease. *CAD* coronary artery disease, *CHD* coronary heart disease, *CI* confidence interval, *CVD* cardiovascular disease, *OR* odds ratio, *RR* relative risk

The main result of this study is that genetic variants predisposing to higher serum magnesium levels may confer a decreased risk of CAD. A genetically predicted 0.1-mmol/L (about one SD) increase in serum magnesium levels was associated with 12% lower odds of CAD in the primary analysis. This finding corroborates the results from observational prospective studies showing inverse associations of circulating magnesium levels and dietary magnesium intake with risk of coronary heart disease and cardiovascular disease [18] (Fig. 3).

There is no gold standard MR analysis method. Available methods have advantages and limitations that balance precision and adjustment for bias. In the present study, several MR approaches were applied to evaluate the robustness of the causal association between serum magnesium levels and CAD. Although we cannot entirely rule out pleiotropy, we observed a consistent inverse association between serum magnesium levels and CAD in conventional MR analysis and sensitivity analyses using the weighted median and heterogeneity-penalised model averaging methods. MR-Egger analysis, which has lower statistical power compared with the other methods, suggested no bias due to pleiotropy (i.e. when a genetic variant affects more than one phenotype) and did not detect a causal association, but the confidence interval was wide. The *I*^2^_GX_ and *F*-statistics were high, suggesting that violation of the NOME assumption was limited and that weak instrument bias due to dilution did not materially affect the results. As in any MR study, we cannot entirely exclude population stratification as a source of bias in this study. However, the GWAS datasets used for the present analyses largely comprised individuals of European ancestry and adjustment was made for ancestry within the contributing studies, reducing possible bias due to population stratification.

There are several plausible mechanisms whereby magnesium may affect the risk of CAD. Magnesium is involved in blood pressure regulation and in glucose and insulin metabolism [1, 2]. Meta-analyses of randomised controlled trials have shown that magnesium supplementation may modestly reduce blood pressure [8, 10–12], fasting glucose [12, 14] and insulin resistance [15]. However, we found no evidence that genetically higher magnesium levels were associated with lower blood pressure or glycaemic traits, suggesting that these risk factors are not likely mediators or confounders of the magnesium-CAD relationship. In addition, the inverse association between serum magnesium levels and CAD is unlikely explained by major lipids, as genetically higher magnesium levels were not associated with cholesterol but were weakly associated with higher triglycerides, which increase CAD risk [37].

Magnesium could potentially confer protection against CAD by enhancing endothelium-dependent vasodilation and reducing vascular resistance, oxidative stress and oxidised lipids, inflammation and thrombosis, and by anti-arrhythmic effects [2, 4, 7, 16, 17]. Several [8, 9, 13] but not all [38, 39] randomised trials have shown that magnesium supplementation improves endothelial function and reduces arterial stiffness. The inconsistent results may be related to magnesium status among study participants, as improvement in endothelial function with magnesium supplementation was observed in trials involving patients with low serum magnesium levels [9] and patients using diuretics [8], which often cause hypomagnesaemia. Both extracellular and intracellular free magnesium can modulate vascular smooth muscle tone [2]. Extracellular magnesium is considered to be a calcium antagonist, because it inhibits many of the physiological actions of calcium [2, 40]. Magnesium decreases calcium release from and into the sarcoplasmic reticulum and protects the cells against calcium overload during myocardial ischaemia [2, 40]. Multiple lines of evidence indicate that a modestly elevated serum calcium level increases CAD risk [41–43]. In this context, mutations in *TRPM6* (encoding a transient receptor potential cation channel) cause hypomagnesaemia with secondary hypocalcaemia [44, 45]. Hence, the observed positive association between the magnesium-raising allele of the genetic variant in *TRPM6* and CAD might be mediated by calcium. Another magnesium-associated genetic variant is located nearby the *ATP2B1* gene, which encodes plasma-membrane calcium ATPase responsible for removal of calcium ions from cells [22].

A limitation of this study is that the specific biological functions of most of the genetic variants associated with serum magnesium levels are unknown (Additional file 1: Table S2). However, the magnesium-associated SNPs have shown association with hypomagnesaemia and with phenotypes related to serum magnesium levels, such as fasting glucose (SNP in *MUC1*), bone mineral density (SNPs in *MUC1* and *TRPM6*) and kidney function (SNPs in *SHROOM3* and *DCDC5*) [22]. Kidney function has been associated with cardiovascular disease risk in observational studies [46], but there was little support for a causal association between kidney function and coronary heart disease in a recent MR analysis [47], suggesting that the observed association between magnesium levels and CAD in the present study is unlikely mediated by kidney function. Further research is needed to better understand the role of the genetic variants and their link to circulating and intracellular magnesium levels.

## Conclusions

This study based on genetics provides evidence that serum magnesium levels are inversely associated with risk of CAD. Randomised controlled trials elucidating whether magnesium supplementation reduces the risk of CAD are warranted. As magnesium supplementation is expected to be most beneficial in individuals with an inadequate magnesium status, such a trial may preferably involve a setting with persons at higher risk of hypomagnesaemia.

## Additional file

Additional file 1: **Table S1.** Association of genetically predicted 0.1-mmol/L increase in serum magnesium with coronary artery disease in inverse-variance weighted and sensitivity analyses. **Table S2.** Genes located in or near the loci for serum magnesium and their biological roles. (DOCX 56 kb)

## Abbreviations

CAD: Coronary artery disease
CI: Confidence interval
GWAS: Genome-wide association study
NOME: NO Measurement Error
OR: Odds ratio
SD: Standard deviation
SIMEX: Simulation extrapolation
SNP: Single-nucleotide polymorphism
