# Supplementary material for: Serum magnesium levels and risk of coronary artery disease: Mendelian randomisation study
Source: BMC Med. 2018 May 17;16:68. doi: 10.1186/s12916-018-1065-z (PMC5956816; doi:10.1186/s12916-018-1065-z)
Supplement: Supplementary file 1 — Table S1. Association of genetically predicted 0.1-mmol/L increase in serum magnesium with coronary artery disease in inverse-variance weighted and sensitivity analyses. Table S2. Genes located in or near the loci for serum magnesium and their biological roles. (DOCX 56 kb) [file 12916_2018_1065_MOESM1_ESM.docx]

**Additional file 1**

Serum magnesium levels and risk of coronary artery disease: Mendelian randomization study

*Authors:* Susanna C. Larsson, Stephen Burgess, Karl Michaëlsson

| **Supplemental table** | **Page** |
| --- | --- |
| **Table S1.** Association of genetically predicted 0.1 mmol/L increase in serum magnesium with coronary artery disease in inverse-variance weighted and sensitivity analyses | 2 |
| **Table S2.** Genes located in or near the loci for serum magnesium and their biological roles | 3 |

**Table S1.** Association of genetically predicted 0.1 mmol/L increase in serum magnesium with coronary artery disease in inverse-variance weighted and sensitivity analyses

|  | **All six SNPs**^a^ | |  | **Excluding rs11144134**^b^ | |
| --- | --- | --- | --- | --- | --- |
| **Method** | **OR (95% CI)** | ***P*** |  | **OR (95% CI)** | ***P*** |
| Inverse-variance weighted, fixed-effects | 0.88 (0.78-0.99) | 0.03 |  | 0.82 (0.72-0.93) | 0.002 |
| Inverse-variance weighted, random-effects | 0.88 (0.74-1.05) | 0.14 |  | 0.82 (0.72-0.93) | 0.002 |
| Weighted median | 0.84 (0.72-0.98) | 0.03 |  | 0.82 (0.71-0.96) | 0.02 |
| Heterogeneity-penalized model averaging | 0.83 (0.71-0.96) | 0.02 |  | 0.83 (0.71-0.95) | 0.008 |
| MR-Egger regression^c^ | 1.19 (0.72-1.98) | 0.50 |  | 0.95 (0.63-1.43) | 0.80 |
| MR-Egger regression, SIMEX method | 1.19 (0.58-2.42) | 0.64 |  | 0.94 (0.23-3.85) | 0.93 |

CI, confidence interval; OR, odds ratio; SIMEX, simulation extrapolation; SNPs, single-nucleotide polymorphisms.

^a^*P* value for heterogeneity between estimates from the six individual SNPs was 0.06.

^b^*P* value for heterogeneity between estimates from the five individual SNPs was 0.74.

^c^MR-Egger regression provided no evidence of pleiotropy; MR-Egger intercept was -0.023 (*P* = 0.21) in the analysis of all six SNPs and -0.010 (*P* = 0.46) in the analysis excluding rs11144134 in the *TRPM6* gene, which was responsible for the observed heterogeneity among estimates from individual SNPs.

**Table S2.** Genes located in or near the loci for serum magnesium and their biological roles

| **SNP** | **Closest gene** | **Biological role** |
| --- | --- | --- |
| rs4072037 | *MUC1* | *MUC1*encodes mucin 1, which is a membrane-bound protein that is a member of the mucin family. Mucins play an essential role in forming protective mucous barriers on epithelial surfaces and are involved in intracellular signaling |
| rs7965584 | *ATP2B1* | *ATP2B1* encodes plasma-membrane calcium ATPase 1 which is responsible for removal of calcium ions from cells |
| rs3925584 | *DCDC5* | *DCDC5* encodes doublecortin domain containing 5 which plays a role in intracellular signal transduction |
| rs11144134 | *TRPM6* | *TRPM6*encodes a TRP ion channel subunit, which is responsible for transcellular magnesium transport by mediating magnesium reuptake at the apical membrane of renal epithelial cells in the distal tubule |
| rs13146355 | *SHROOM3* | *SHROOM3* encodes a PDZ-domain-containing protein, which may be involved in regulating cell shape in certain tissues |
| rs448378 | *MDS1* | *MDS1* encodes myelodysplasia syndrome 1 which has unknown function |

SNP, single-nucleotide polymorphism.
